# An Empirical Mode Decomposition Fuzzy Forecast Model for Air Quality

**DOI:** 10.3390/e24121803

**Published:** 2022-12-09

**Authors:** Wenxin Jiang, Guochang Zhu, Yiyun Shen, Qian Xie, Min Ji, Yongtao Yu

**Affiliations:** Faculty of Computer and Software Engineering, Huaiyin Institute of Technology, Huai’an 223003, China

**Keywords:** air quality, empirical mode decomposition, extreme learning machine, adaptive fuzzy inference system

## Abstract

Air quality has a significant influence on people’s health. Severe air pollution can cause respiratory diseases, while good air quality is beneficial to physical and mental health. Therefore, the prediction of air quality is very important. Since the concentration data of air pollutants are time series, their time characteristics should be considered in their prediction. However, the traditional neural network for time series prediction is limited by its own structure, which makes it very easy for it to fall into a local optimum during the training process. The empirical mode decomposition fuzzy forecast model for air quality, which is based on the extreme learning machine, is proposed in this paper. Empirical mode decomposition can analyze the changing trend of air quality well and obtain the changing trend of air quality under different time scales. According to the changing trend under different time scales, the extreme learning machine is used for fast training, and the corresponding prediction value is obtained. The adaptive fuzzy inference system is used for fitting to obtain the final air quality prediction result. The experimental results show that our model improves the accuracy of both short-term and long-term prediction by about 30% compared to other models, which indicates the remarkable efficacy of our approach. The research of this paper can provide the government with accurate future air quality information, which can take corresponding control measures in a targeted manner.

## 1. Introduction

Medical research shows that air has a direct effect on the blood circulation, immunity, cardiovascular system, and nervous system of the human body. Air pollution has a variety of adverse effects, both physiological and psychological, on people’s health. Inhaling dirty air for a long time can cause many physical and psychological problems, such as dizziness, chest tightness, difficulty concentrating, and even induce some diseases. At the same time, severe air pollution can also greatly reduce visibility, make it difficult for people to travel, and even cause traffic accidents. Air pollution also has an impact on the ecological environment. It can damage the diversity and stability of the natural ecosystem structure. In addition, severe air pollution can cause physical damage to buildings, affecting the appearance of buildings and their service lives.

Since the first industrial revolution, industrialization and urbanization use a large number of resources, resulting in increased exhaust emissions and plummeting air quality, which brings unprecedented negative effects to people’s living environment. At the same time, there are more and more research works on air quality in academic circles. The prediction of the Air Quality Index (AQI) is undoubtedly an important link among them.

There are many prediction methods for air quality. The earliest prediction method in the world is potential prediction. However, due to it only considering of meteorological factors, the prediction results are not accurate, so it does not belong to the current mainstream prediction methods. Numerical prediction and statistical prediction are the most popular methods for air quality prediction in the world. Numerical prediction is a method based on physics and combined with the knowledge of many subjects. It has good accuracy, timeliness, and efficiency. However, this method is very difficult, needing accurate data support. The problems, such as the large amount of calculation, make it only suitable for large institutions or government agencies. Different from numerical forecasting, statistical forecasting does not need to understand the development law of air quality and the relationship between various factors. It is a method that only needs to analyze the law and make predictions through data. It is suitable for institutions, governments, as well as individuals. With its obvious advantages, statistical prediction has received more and more attention in the research of air quality prediction. Common statistical forecasting methods include the grey model, clustering, and neural networks.

By analyzing and determining the relationship between the concentration of air pollutants and environmental factors, clustering can obtain the correlation between each element, to predict the air quality. The concentration of air pollutants is characterized by nonlinear and non-stationary characteristics, including high noise levels and outliers. To solve this problem, Chen et al. took PM${}_{2.5}$ as an example, decomposed PM${}_{2.5}$ data by ensemble empirical mode decomposition, selected the most effective set of data, and projected it by the least-squares method [1]. Clustering can not only measure the factors that affect the concentration of air pollutants, but also improve the prediction accuracy and reduce the time complexity. Lee et al. clustered the spatial heterogeneity of PM${}_{2.5}$ concentration and used a mixed-effects model to make aerosol optical thickness PM${}_{2.5}$ concentrations of robust predictors and through the remote sensing map to predict the concentration of PM${}_{2.5}$ [2]. Mahajan et al. clustered the geographic distances of 557 urban stations in Taiwan and used a mixed model to make predictions. The experiment showed that clustering based on geographical distance can reduce the prediction error rate and also reduce the calculation time [3]. Xiao et al. used clustering to divide the Chinese space into seven regions. Each region was trained by random forest, the generalized additive model and limit gradient lifting, and finally, the additive set model was used to combine the prediction [4].

The grey model (GM) can be processed according to fewer time series data and extract effective information from known information. It is a first-order model with only a single variable, and its prediction curve is relatively simple. Wu et al. proposed a grey Holt–Winters model, which used the grey cumulative generation to deal with the unstable air quality data and the Holt–Winters method to deal with the seasonal effects, with high prediction accuracy [5]. Xiong et al. proposed the nonlinear multivariable GM(1,N) model based on interval grey number sequence. It deduces the upper and lower limits of the interval gray number according to the kernel formula and gray radius to simulate and predict [6]. With fewer data, the grey model can predict very well. However, with a large amount of data, the effect of the grey model is not ideal. Other methods of machine learning (ML) can better solve this problem. Zakeviciute et al. used traffic vehicle flow data to predict air quality data through the decision tree [7]. The experiment showed that the prediction accuracy is improved when the traffic flow is large in the daytime, and it is suitable for countries with a smalleconomy. Gu et al. proposed a heuristic recursive air quality forecast model [8]. It extracts air quality data and meteorological data and makes cycle prediction with the support vector machine. Castelli et al. improved the regression of the support vector machine by taking the radial basis function as the core of the support vector regression machine [9]. The improved support regression machine was used to predict air quality and achieved good results. Lyu et al. proposed a bias-correction model, including feature selection, clustering, error estimation, and interpolation [10]. The model uses the historical relationship among the predicted variables, the observed variables, and the model bias to make the current forecast. Liu et al. proposed an n-step recursive prediction model based on Seq2Seq to predict air quality [11]. It replaces the encoder in Seq2Seq with a fully connected encoder to speed up the training process. At the same time, combined with the position embedding technology, the fully connected encoder can discover the timing relationship between the sequences. Some methods for other task prediction are helpful for air quality prediction. Ma et al. proposed a hybrid approach integrating k-fold cross-validation (CV), metaheuristic support vector regression (SVR), and the nonparametric Friedman test, which aims to enhance the repeatability of predictions [12]. In order to enhance the reliability of geohazard prevention using ML models, Ma et al. proposed a systematic framework based on k-fold CV, MHs, and SVR [13].

Deep learning is also widely used in air quality prediction due to its powerful nonlinear fitting ability. Ray et al. proposed a prediction model based on deep learning [14]. It uses correlation analysis to find the most appropriate feature set, which is used to predict air quality. Qi et al. embedded feature selection and semi-supervised learning in different levels of the deep learning network and integrated air quality interpolation, prediction, and feature analysis into a model [15]. Yang et al. proposed a hybrid model combining complementary integrated empirical mode decomposition, improved cuckoo search, the differential evolution algorithm, and the Elman neural network to predict air quality [16]. Cheng et al. used a recursive neural network to extract information from the input sequence and measure the impact of other sites, using the fully connected layer to obtain the predicted results [17]. Yi et al. considered the spatial correlation of air pollutants, processed sparse air quality data with spatial transformation components, and fused the data affecting air quality of multiple cities with neural distribution structures [18]. Li et al. proposed an air quality prediction method based on spatiotemporal deep learning, which extracted inherent air quality characteristics with stacked autoencoder models and trained them in a greedy layer-by-layer manner. It can simultaneously predict air quality at multiple sites and shows temporal stability over the seasons [19]. Peng et al. proposed a learning method based on an extremum learning machine, which learns continuously as the data increase, combined with multiple linear regressions and multi-layer perceptron networks, to predict air quality [20]. Kabi et al. combined the confidence expert system with deep learning and optimized the confidence expert system so as to find the nonlinear dependence among the relevant variables, so as to predict air quality [21]. Bai et al. proposed a seasonal stacked automatic encoder model combining seasonal analysis and deep feature learning [22]. The Kendall correlation coefficient method was used to explore the internal relationship between air quality and seasons. A deep neural network was used to extract features, and a regression stacked automatic encoder was used to conduct the prediction. Pardo et al. used long short-term memory (LSTM) to predict air quality over the next 12 and 24 h [23]. Athira et al. used a variety of deep learning methods to predict the air quality, including recursive neural networks, LSTM, and gated recursive units [24]. Soh et al. combined an artificial neural network, convolutional neural network, and LSTM to extract spatiotemporal relations [25]. That is, the trend extracted from the correlation between the adjacent positions and the similar positions in the time horizon was applied to predict air quality. Zhou et al. proposed a deep multi-output model based on LSTM [26]. The model combines three deep learning algorithms, discrete neurons, gradient descent, and L2 regularization, to extract the key factors of the spatiotemporal relationship and reduce the error accumulation and propagation in multi-step advanced air quality forecast. Lin et al. proposed an air quality prediction system based on neural networks [27]. Historical time series data were used to derive a set of fuzzy rules or neural fuzzy networks to predict future air pollutant concentrations and environmental factors. Yan et al. used the deep learning network model based on spatiotemporal clustering to establish a multi-time and multi-site prediction model for air quality in Beijing. Compared with back-propagation (BP), the model has a good prediction effect [28]. Wang et al. proposed a deep space–time integration model [29]. The model adopts the division strategy based on weather patterns for clustering and generates the spatial data of relative stations and relative regions by analyzing the Granger causality between stations, so as to find the spatial correlation. Finally, the LSTM forecasts different types of data to understand the long-term and short-term correlation of air quality. A CT-LSTM method was proposed by Wang et al., which combines the chi-squared test (CT) and LSTM network model to establish a prediction model [30]. R. Janarthanan et al. proposed a deep learning model for classifying AQI values, based on SVR and LSTM [31].

From the current research status at home and abroad, most methods focus on the impact of a few pollutants such as PM${}_{2.5}$ and fail to fully consider the impact of other air data on air quality. Although some deep learning models using back-propagation have good prediction accuracy, they easily fall into a local optimum during the training process, and the large number of iterative calculations causes their convergence speed to be too slow. In order to solve the above problems, we propose an empirical modal decomposition fuzzy prediction model of air quality based on extreme learning machine (ELM) for air data containing multiple pollutants. In this paper, the historical data of air quality of a single city are analyzed to explore the internal laws, so as to predict air quality.

To summarize, our work makes the following contributions:

1. We propose an empirical-mode-decomposition-based fuzzy prediction model for air quality, which can effectively explore the internal rules of air quality data and provide more accurate support for prediction;
2. The model uses the adaptive network-based fuzzy inference system to fuse the prediction results obtained by the extreme learning machine to obtain the prediction results with high precision and good generalization performance;
3. We collect and preprocessed a new dataset that contains much information about local pollution control factors.

## 2. Materials and Methods

### 2.1. Data Set

**Figure 1:**
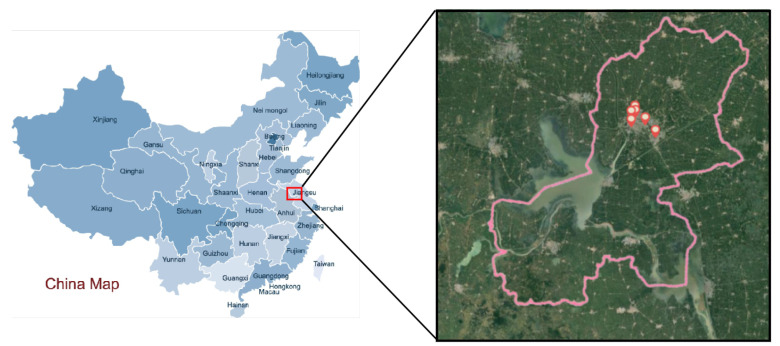
The locations of the air quality monitoring stations in the Huaian region (red marks).

In this paper, the air quality of Huaian in Jiangsu province was selected for prediction. As shown in Figure 1, the six monitoring stations almost cover the living areas of Huaian city. Therefore, air pollutant data measured by them can better represent the living environment of the people of Huaian. Huaian has several advantages: Huaian is located on the north–south boundary of China, and it is affected by the monsoon climate. Climate change is obvious, and the variation trend of air pollutant concentration is obvious; thus, it is a good representative; the economic development is good, with more and accurate air quality detection points; the terrain is mainly plains and partly hilly; there are many rivers in the territory, and there is a large lake. The environment is changeable, and there are many accurate data, so it has a high research value. Therefore, the selection of Huaian air quality data for the experiment of the method can show whether the method has good learning ability and prediction accuracy in the face of complex and changeable situations.

**Figure 2:**
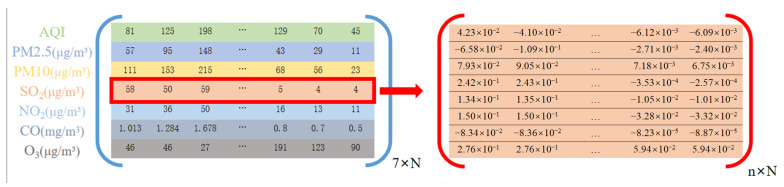
Data form. The matrix on the left is 7 factors of the original data, with a total of *N* days; the matrix on the right is the concentration of a pollutant’s concentration (taking SO${}_{2}$ as an example) after normalization and decomposition.

The data came from a data center of the China Ministry of Ecology and Environment (http://datacenter.mee.gov.cn, accessed on 5 February 2021), collected by day. The data provided by the monitoring stations are AQI, PM${}_{2.5}$, PM${}_{10}$, sulfur dioxide (SO${}_{2}$), nitrogen dioxide (NO${}_{2}$), carbon monoxide(CO), and ozone (O${}_{3}$), of which the measurement unit of PM${}_{2.5}$, PM${}_{10}$, SO${}_{2}$, NO${}_{2}$, and O${}_{3}$ is (µg/m${}^{3}$), while CO is measured in (mg/m${}^{3}$). The data are shown in Figure 2. The detection time of SO${}_{2}$, NO${}_{2}$, CO, and O${}_{3}$ is measured in hours, while PM${}_{2.5}$ and PM${}_{10}$ are the average concentrations during a 24 h period. For the convenience of the statistics and research, all main pollutants are expressed in 24 h average concentrations in this paper. The data are highly accurate and scientific, which is of great significance to the algorithm prediction experiment.

### 2.2. Empirical Mode Decomposition

**Figure 3:**
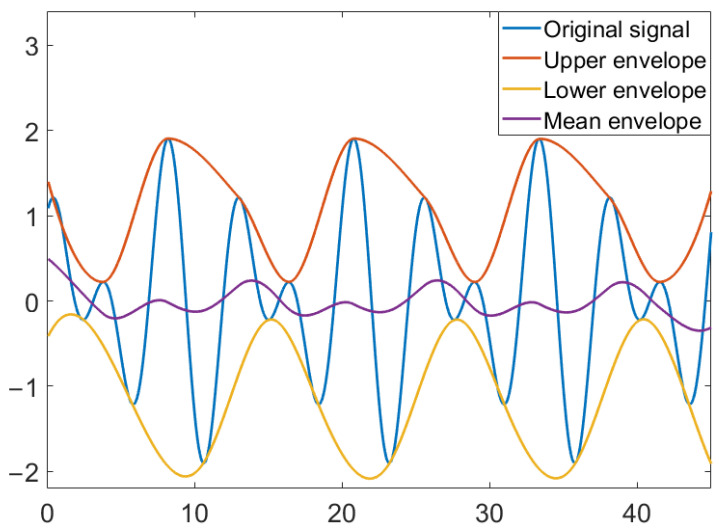
EMD schematic diagram.

Empirical mode decomposition (EMD) is an adaptive signal analysis method proposed by Huang et al. [32]. Different from the general signal analysis method, it does not need to set the basis function. Instead, by analyzing the trend of the signal itself in the characteristic time scale, the original signal is decomposed into sub-signals of different frequencies. This is a method with a wide range of applications and suitable for all kinds of signal analysis. The EMD first smooths the signal and then decomposes the trend of different characteristics in the signal at time scales. The decomposed sequence of different characteristic time scales is called the intrinsic mode function (IMF). Figure 3 is a schematic diagram of EMD.

EMD is finds the extreme point of the original signal, where the maximum is fit to form the upper envelope and the minimum is fit to form the lower envelope, and then, the mean values of the two envelopes are taken to form the mean envelope.

### 2.3. Extreme Learning Machine

**Figure 4:**
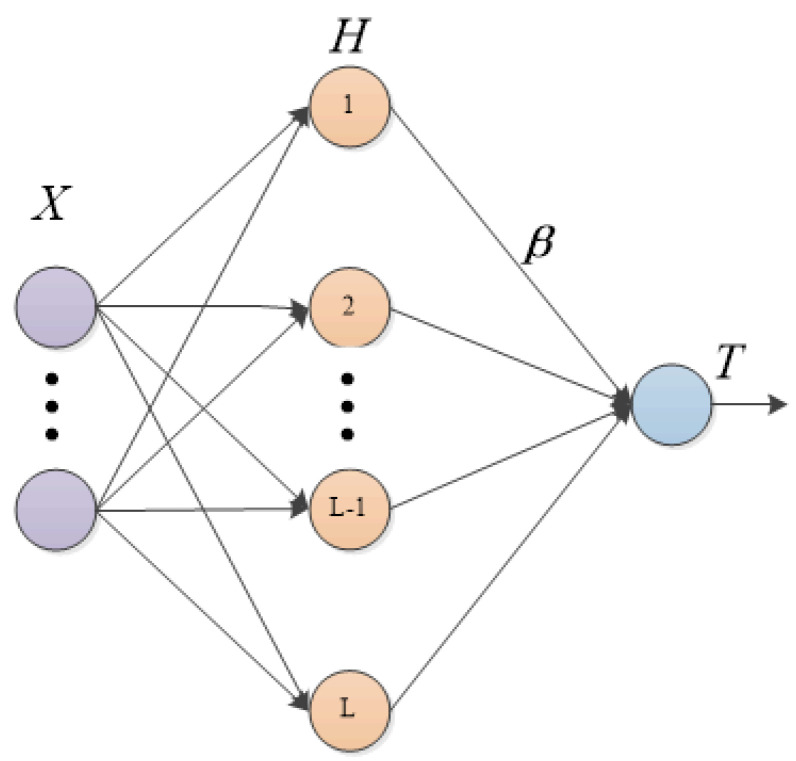
ELM structure diagram.

Models such as the BP neural network and the convolutional neural network are widely used in air quality prediction tasks [33]. However, the BP algorithm in the above models makes it very easy for them to fall into a local optimum during the training process, and the large number of iterative calculations causes their convergence speed to be too slow. Extreme learning machine is an improved single-hidden-layer feedforward neural network proposed by Huang Guangbin [34] in 2004. Compared with other time series prediction ML models, ELM shows a faster learning speed and better generalization performance [35]. Figure 4 is the schematic diagram of ELM. Different from other neural networks, ELM’s training process is extremely simple. The number of hidden nodes can be set, and the optimal solution can be obtained after one training. Compared with the traditional gradient algorithm, ELM has the advantage of an extremely fast learning speed and solving the problems such as overfitting and local minima. Meanwhile, its generalization ability performs better in some applications.

Suppose the excitation function of an ELM is g(x) and there are *N* samples and *L* hidden layer nodes. The input is *X*; the expected output is *T*; β is the output weight of the hidden layer. For a set of inputs $x_{i}$, given the corresponding expected outputs $t_{i}$, input layer nodes to hidden layer input weight $a_{i}$, bias $b_{i}$, and hidden layer output weight $\beta_{i}$, then:

(1) $$\sum_{i=1}^{L}g\left(x_{i}a_{i}+b_{i}\right)\beta_{i}=t_{i}$$

*H* is the output matrix of the hidden layer, then:

(2) $$H={\left[\begin{array}{c} g(x_{1}a_{1}+b_{1})\\ \cdots \\ g(x_{N}a_{N}+b_{N}) \end{array}\right]}_{N*L}$$

The output of ELM can be denoted as the following matrix:

(3) Hβ=T

Since $a_{i}$ and $b_{i}$ in ELM are randomly determined, *H* is also determined, so the training process of ELM is actually the process of solving β, then:

(4) $$\beta =H^{-}T$$

### 2.4. Adaptive Network-Based Fuzzy Inference System

The adaptive network-based fuzzy inference system (ANFIS) is a kind of neural fuzzy inference system proposed by J. S. R. Jang [36]. The ANFIS is widely used in various fields due to its advantages of convenience, high efficiency, and wide adaptability. If the ANFIS has two inputs $e_{1}$ and $e_{2}$ and one output *y*, the rule library has the following rules:

(1) If $e_{1}$ is $A_{1}$ and $e_{2}$ is $B_{1}$, then $y=p_{1}e_{1}+q_{1}e_{2}+z_{1}$;
(2) If $e_{1}$ is $A_{2}$ and $e_{2}$ is $B_{2}$, then $y=p_{2}e_{1}+q_{2}e_{2}+z_{2}$.

**Figure 5:**
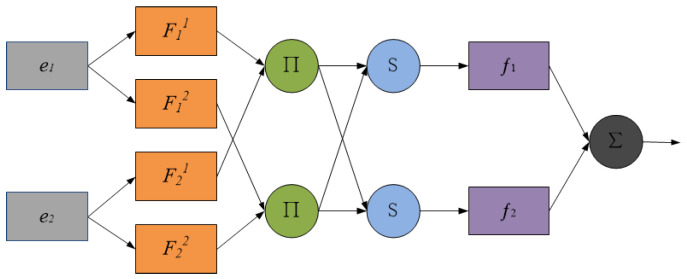
The ANFIS structure diagram.

The typical ANFIS structure is shown in Figure 5, with a total of five layers. The first layer is the membership function layer of the input variables, which is responsible for the fuzzification of the input signals, where $e_{1}$ and $e_{2}$ are the inputs, $F_{i}^{j}$ is the fuzzy set, representing the *j*-th rule of $e_{i}$, and the output result represents the degree to which the input $e_{i}$ is subordinated to $F_{i}^{j}$. The second layer is used to release the rules; the strength of the layer in the figure with ∏ is multiplied by the input signal and its product output. The third layer is the normalization layer of the rule strength, represented by N in the figure, which is responsible for calculating the normalized credibility of rule *j* of $e_{i}$. The fourth layer is the output layer of the fuzzy rules, and each node of this layer is the adaptive node. The fifth layer is a fixed node that calculates the total output of all the input signals.

### 2.5. Empirical-Mode-Decomposition-Based Fuzzy Prediction Model

**Figure 6:**
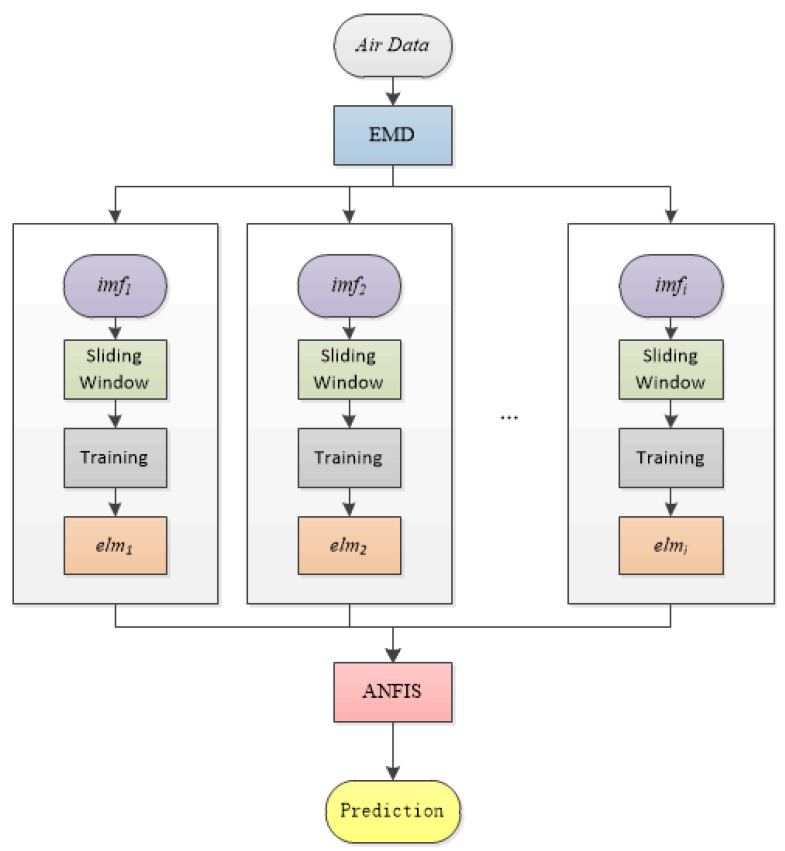
The EMD-FPM structure diagram.

The forecast flow chart of the empirical-mode-decomposition-based fuzzy prediction model (EMD-FPM) is shown in Algorithm 1. The EMD-FPM decomposes the concentration of air pollutants to obtain the variation trend of the concentration of air pollutants in different time scales, which is the IMF. Then, the IMF is trained with ELM, and the ANFIS is used to fit the training results to obtain the final prediction value. The structure diagram is shown in Figure 6.
**Algorithm 1:** Empirical mode decomposition fuzzy forecast model **Input**: Airdata; **Output**: Predicted value *y* of AQI from day t+1 to day t+c1:*A* = pretreat(Airdata);2:$IMF=(imf_{1},imf_{2},\ldots ,imf_{i},\ldots ,imf_{n})$ = EMD(*A*);3:**for** i = 1:n4:   $x_{i}$ = win($imf_{i}$);5:   ei = elmi($x_{i}$);6:**End**7:$e=(e_{1},e_{2},\ldots ,e_{i},\ldots ,e_{n})$;8:*y* = ANFIS(*e*)

**Figure 7:**
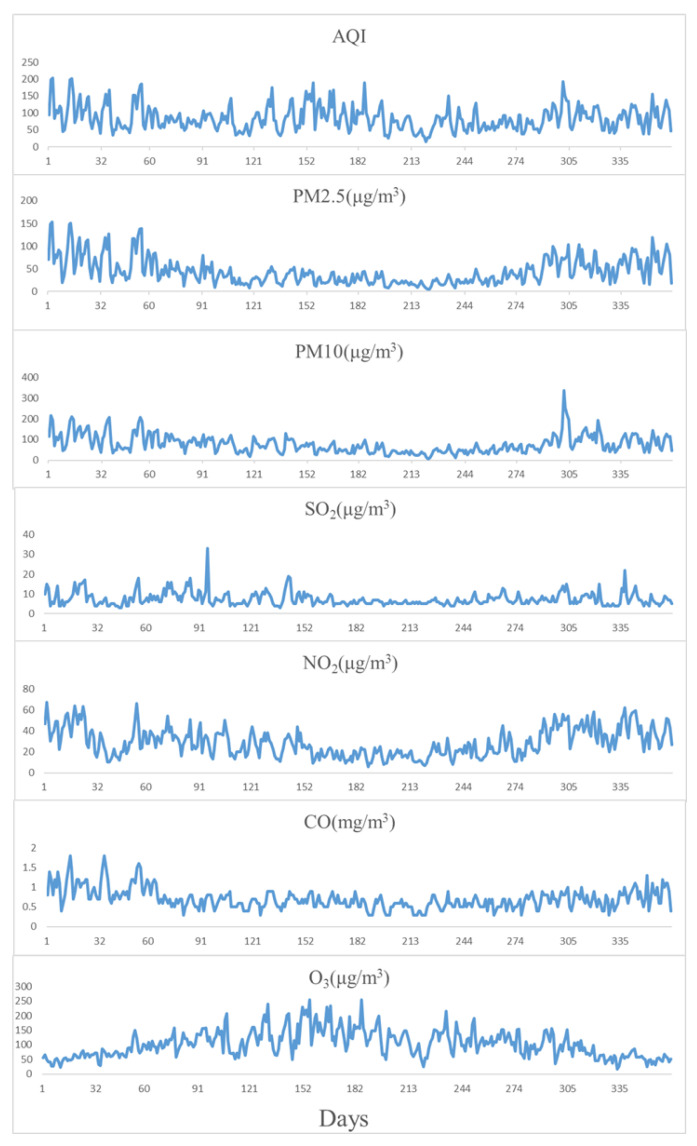
Changes in 7 factors of air data in 2019.

For example, the task is to predict the AQI from 1 January 2020 to 5 January 2020 by inputting the air data for the whole year of 2019. The average value of each indicator in the whole day’s data is input into the EMD-FPM. As shown in Table 1 and Figure 7, the values of PM${}_{10}$ and O${}_{3}$ fluctuate violently in the main pollutants throughout the year, while the values of SO${}_{2}$ and CO fluctuate gently. After the EMD-FPM’s prediction, the output result is a matrix (7×5) vector with the AQI and the other six main pollutants from 1 January 2020 to 5 January 2020.

In this paper, the EMD-FPM is described as follows, taking carbon monoxide (CO) as an example:

(1) The CO concentration data of the air pollutant are taken as the input and normalized to obtain CO(t), where *t* is the number of days.

(2) EMD is used to process the data to obtain the corresponding IMF. The specific steps are as follows:

First, find all the maximum and minimum points of CO(t), and then, fit all the maximums and all the minimums through function fitting to obtain the upper envelope up(t) and the lower envelope low(t). $m_{1}\left(t\right)$ is the mean envelope of the original signal, which is the mean of the upper and lower envelopes:

(5) $$m_{1}\left(t\right)=\frac{up(t)+low(t)}{2}$$

Subtract $m_{1}\left(t\right)$ from CO(t), and obtain a new signal $h_{1}^{1}\left(t\right)$ with the low frequencies removed:

(6) $$h_{1}^{1}\left(t\right)=CO\left(t\right)-m_{1}\left(t\right)$$

In fact, CO(t) is complex and not regular. The calculated results generally do not meet the IMF conditions, so the above steps need to be repeated. Assume that $h_{1}^{k}\left(t\right)$ meets the conditions of the IMF after repeating the above steps *k* times, then the first-order IMF component of the original signal is:

(7) $$imf_{1}\left(t\right)=h_{1}^{k}\left(t\right)$$

The original signal x(t) is subtracted by $imf_{1}\left(t\right)$ to obtain a new signal $r_{1}\left(t\right)$ with the high frequency component removed:

(8) $$r_{1}\left(t\right)=x\left(t\right)-imf_{1}\left(t\right)$$

Repeat the above process for $r_{1}\left(t\right)$ to obtain a second IMF component $imf_{2}\left(t\right)$, and so on, until the end, and the IMF with *n* times of decomposition is obtained, $IMF=(imf_{1},imf_{2}$,…, $imf_{i},\ldots ,imf_{n})$.

(3) The sliding window of size *c*+1 is used to slide IMF. The top *c* values of each sliding window are the input to ELM, and the (*c*+1)-th value is the output of ELM.

(4) The input and output obtained in Step (3) are trained with the ELM to obtain $elm_{i}$. Then, the IMF corresponding to the air pollutant concentration is ELM, $ELM=(elm_{1},elm_{2},\ldots ,$$elm_{i},\ldots ,elm_{n})$.

(5) The predicted result is marked as $e_{i}$, then the predicted result corresponding to the ELM is $e=(e_{1},e_{2},\ldots ,e_{i},\ldots ,e_{n})$. The ANFIS is used for fitting, then the first-layer output of the ANFIS is:

(9) $$O_{ij}^{1}=\mu_{F_{i}^{j}}\left(e_{i}\right)$$

where the membership function $\mu_{F}$ is determined by a number of parameters, which are called antecedent parameters.

The output of the second layer is as follows:

(10) $$O_{j}^{2}=\omega_{j}=\prod \mu_{F^{j}}\left(e_{i}\right)$$

The output of the third layer is as follows:

(11) $$O_{j}^{3}=\bar{\omega_{j}}=\frac{\omega_{j}}{\sum \omega_{j}}$$

The output of the fourth layer is as follows:

(12) $$O_{j}^{4}=\bar{\omega_{j}}f_{j}=\bar{\omega_{j}}\left(\sum p_{i}e_{i}+q_{i}\right)$$

The output of the fifth layer, that is the final prediction result, is as follows:

(13) $$O^{5}=\sum \bar{\omega_{j}}f_{j}$$

The final prediction results fit the predicted values of different time scales. The model has a good prediction effect for the data of a single city site.

### 2.6. Evaluation Standard

In order to evaluate the accuracy of the prediction, the following evaluation criteria were selected in this paper:

(1) The mean-squared error (MSE) is the most commonly used evaluation standard to test the error between the predicted value and the true value. The formula is as follows:

(14) $$MSE=\frac{\sum {\left(y-y^{\prime }\right)}^{2}}{N}$$

(2) The mean absolute error (*MAE*), namely the mean of absolute error, is used to reflect the actual situation of the predicted value error. The formula is as follows:

(15) $$MAE=\frac{\sum |y-y^{\prime }|}{N}$$

(3) The standard deviation (*SD*), the arithmetic square deviation of variance, is used to reflect the dispersion between the predicted value or the true value and the mean value. Taking the true value as an example, the formula is as follows:

(16) $$SD=\sqrt{\frac{\sum {\left(y-\bar{y}\right)}^{2}}{N}}$$

where $y^{\prime }$ represents the predicted value, *y* represents the true value, $\bar{y}$ represents the average value of the true value, and *N* represents the number of predicted or true values.

## 3. Results and Discussions

### 3.1. Contrast Experiment

Three groups of comparative experiments were selected for the experiment: ELM, back-propagation (BP) neural network, and nonlinear auto regressive (NAR) neural network, as follows:

(1) The BP neural network is a multi-layer feedforward neural network [37], which is widely used in many research fields. The training process of the BP neural network is divided into two stages. First, the data are propagated forward from the input layer to the hidden layer and then to the output layer. The error is then propagated back from the output layer to the hidden layer and then to the input layer.

(2) The NAR neural network [38] is a time series neural network that uses itself as a regression variable, that is a linear combination of random variables at certain moments to describe a nonlinear target moment. The NAR neural network is widely used in various forecasting models and has good results.

### 3.2. Decomposition Result

In this paper, MATLAB was used to implement the algorithm and plot the results. We used a Lenovo computer with an Inter Core i5-6500 3.20 GHz processor, 8 GB RAM, and a Windows 10 Professional 64-bit operating system.

**Figure 8:**
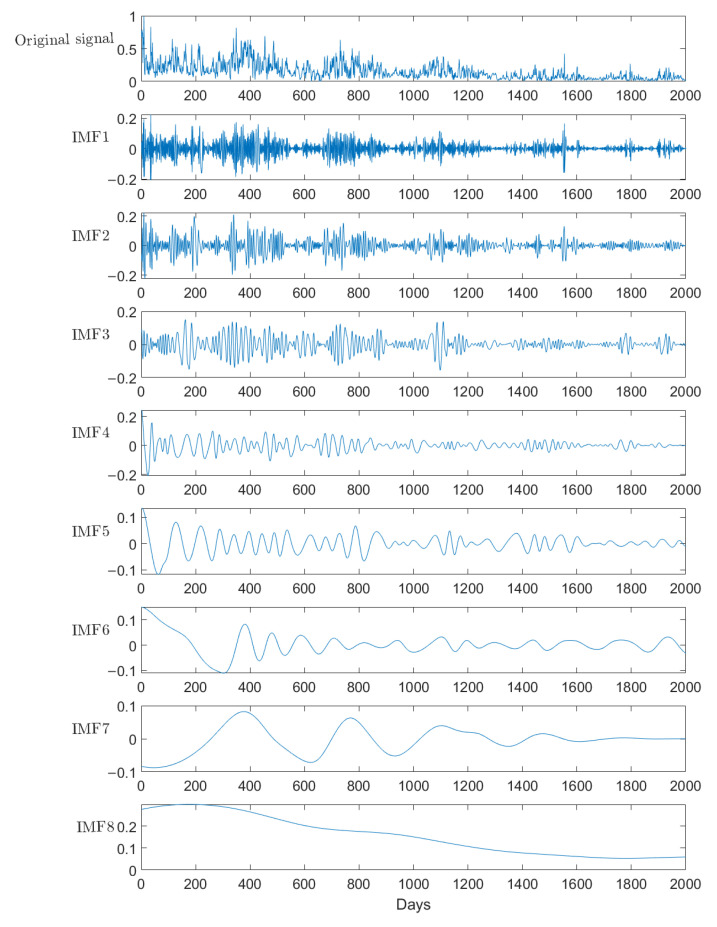
EMD, taking sulfur dioxide as an example.

The SO${}_{2}$ concentration in Huaian was normalized, and EMD was carried out. The original data and decomposition results are shown in Figure 8. The first one is the original data, and the rest are the IMF. As can be seen from the figure, the changes of the original data are complex and diverse without obvious rules, but they show a downward trend over time. This reflects the government’s attention to the effect of air quality control and the public. Through multiple decompositions, the original unstable and non-smooth data gradually become smooth. As can be seen from the top five decomposition, after a period of stable sulfur, there is a period of relatively obvious fluctuations. This is due to the centralized treatment of air quality, which failed to be effectively continued, resulting in the recurrence of pollution. It can be seen that air quality control is a long-term process, and temporary control cannot completely eliminate pollution. It can be seen from the last three decompositions that the overall change trend is fluctuating, and the last one shows a downward trend, just like the overall trend of the original data. It can be also seen that EMD is very effective for trend analysis of the data under different time scales.

### 3.3. Short-Term Prediction

**Figure 9:**
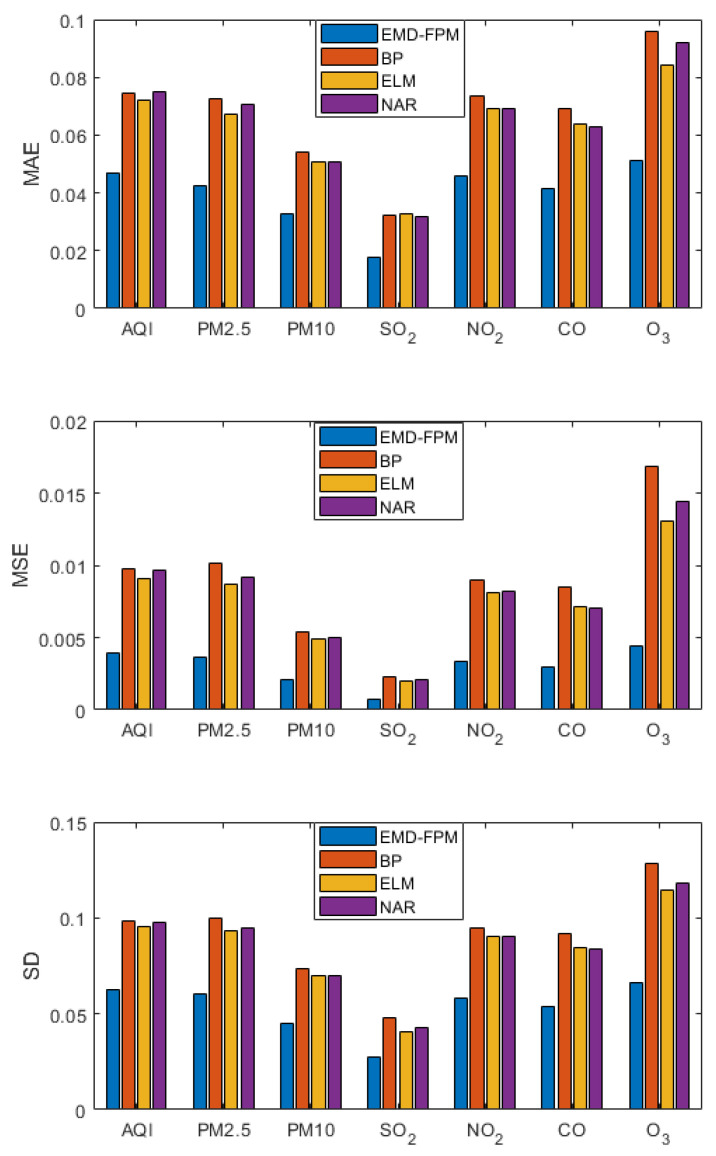
Short-term forecast comparison.

A short-term prediction was made for each index of air quality. That is, the data of the top six days were used to predict the value of the seventh day, and the prediction results are shown in Figure 9. As can be seen from the figure, the BP neural network, ELM, and the NAR neural network have similar performances. These three neural networks only calculate the predicted value of air quality and fail to fully explore the internal relationship of air quality, so the performances are close. ELM has the best effect, and the error range of the three indicators is small. This is because ELM performs better when the data volume is small. The EMD-FPM has good accuracy and generalization performance in the prediction of the Air Quality Index or air pollutants, which is about 30% higher than other algorithms. All three evaluation indexes are superior to the other algorithms, which means that the EMD-FPM algorithm not only has high accuracy, but also has good adaptability to special situations.

### 3.4. Long-Term Prediction

**Figure 10:**
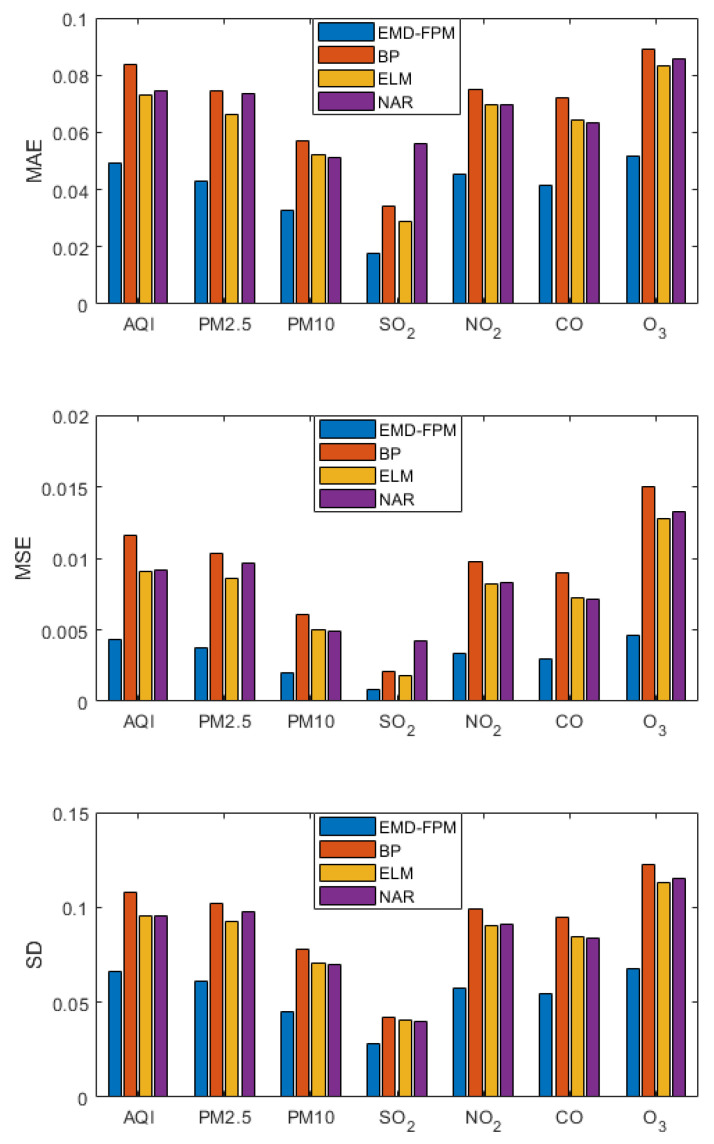
Long-term forecast comparison.

A long-term prediction was made for each indicator of air quality. That is, the data of the first 12 days were used to predict the value from the 13th day to the 15th day, and the prediction results are shown in Figure 10. As shown by the long-term prediction results, the EMD-FPM is far superior compared to the other algorithms. The prediction accuracy is 30% 40% higher than the other algorithms. Compared with the short-term prediction, the error of each evaluation index in all algorithms increased, which is the inevitable result of the long-term prediction. However, the error increase of the EMD-FPM can be ignored, while other algorithms grow significantly. Therefore, the EMD-FPM also has good performance in long-term prediction.

## 4. Conclusions

In recent decades, air pollution has posed major hazards to human health, prompting widespread public concern. Severe air pollution can cause respiratory diseases, while good air quality is beneficial to physical and mental health. The empirical mode decomposition fuzzy forecast model for air quality, which is based on extreme learning machine, was proposed in this paper. Empirical mode decomposition can smooth the complex and changeable air quality data and obtain the change trend of air quality under different time scales. According to the change trend under different time scales, extreme learning machine was used for training, and the corresponding prediction value was obtained. The adaptive fuzzy inference system was used for fitting to obtain the final air quality prediction result. A comparative examination of our developed model with other deep learning models, BP, ELM, and NAR, was also carried out on the Huaian air dataset. The experimental findings indicated that the EMD-FPM is superior to the other models in in both short-term and long-term prediction. The research of this paper can provide the government with accurate future air quality information, which can take corresponding control measures in a targeted manner. This study has certain limitations. The study used a small sample size and focused only on the Huaian region. Future research should add more datasets and multiple regions to compare the findings with our study. Future studies should take into account the impact of air pollution in surrounding cities on the study region. Additionally, it is also important to examine seasonal weather conditions.

## Figures and Tables

**Table 1:** Statistics about air data in 2019.

	AQI	PM${}_{2.5}$	PM${}_{10}$	SO${}_{2}$	NO${}_{2}$	CO	O${}_{3}$
MAX	203	153	336	33	67	1.8	255
MIN	15	5	6	3	6	0.3	17
AVERAGE	84.5	44.1	78.3	7.4	28.8	0.7	102.8
MEDIAN	78.5	36	69	6	26	0.7	98
STANDARD DEVIATION	36.1	29.4	44.5	3.4	13.4	0.3	46.2

## Data Availability

Not applicable.

## Abbreviations

The following abbreviations are used in this manuscript:

AQI	Air Quality Index
GM	Grey model
ML	Machine learning
CV	Cross-validation
SVR	Support vector regression
LSTM	Long short-term memory
BP	Back-propagation
CT	Chi-squared test
ELM	Extreme learning machine
EMD	Empirical mode decomposition
IMF	Intrinsic mode function
ANFIS	Adaptive network-based fuzzy inference system
FPM	Fuzzy prediction model
EMD-FPM	Empirical-mode-decomposition-based fuzzy prediction model
MSE	Mean-squared error
MAE	Mean absolute error
SD	Standard deviation
NAR	Nonlinear auto-regression
